# The Impact of Urinary Incontinence, Sexual Dysfunction, and Depressive Symptoms on Health-Related Quality of Life Over the 12-Month Postpartum Period

**DOI:** 10.1097/jnr.0000000000000706

**Published:** 2025-10-02

**Authors:** Meng-Kuan Chiang, Wei-An Lin, Chien-Nan Lee, Shiow-Ru Chang

**Affiliations:** 1 School of Nursing, College of Medicine, National Taiwan University, Taipei, Taiwan; 2 Center for Advancement of Nursing Education, Koo Foundation Sun Yat-Sen Cancer Center, Taipei, Taiwan; 3 Department of Occupational Medicine, Ten-Chan General Hospital, Taoyuan County, Taiwan; 4 Department of Environmental and Occupational Medicine, National Taiwan University Hospital, Taipei, Taiwan; 5 Department of Obstetrics and Gynecology, National Taiwan University Hospital, Taipei, Taiwan

**Keywords:** postpartum, health-related quality of life, urinary incontinence, sexual dysfunction

## Abstract

**Background:**

The physical and psychological challenges faced by postpartum women adversely affect their health-related quality of life (HRQoL). However, the influence of urinary incontinence (UI), sexual dysfunction, and depressive symptoms on HRQoL across the first postpartum year remains unclear.

**Purposes:**

This study was designed to investigate the association of UI, sexual dysfunction, and depressive symptoms with HRQoL and to examine changes in HRQoL across the initial 12-month postpartum period.

**Methods:**

The participants (*n*=613) completed the study questionnaire at four time points: 4–6 weeks and 3, 6, and 12-months postpartum. The questionnaire was mailed from a medical center maternity unit and included the International Consultation on Incontinence Questionnaire Urinary Incontinence Short Form, Female Sexual Function Index, the Center for Epidemiologic Studies Depression Scale, and the 36-item Short-Form Health Survey.

**Results:**

Moderate to severe UI (β =−2.99), sexual satisfaction (β =−0.43), and lubrication (β =0.44; all *p* <.05) were associated with physical HRQoL over the 12-month postpartum period. Mental HRQoL was influenced by moderate to severe UI (β =−1.3), sexual satisfaction (β =0.61; both *p* <.05), and depressive symptoms (β =−11.07; *p* <.001). The lowest *p*hysical and mental HRQoL scores were identified in the first month postpartum (all *p* <.001). Physical HRQoL increased more significantly at 6 and 12 months than at 3-months postpartum (both *p* ≤.001).

**Conclusions/Implications for practice:**

The first month postpartum represents a critical period for assessing HRQoL, when it is lowest. UI severity, sexual satisfaction, lubrication, and depressive symptoms were all shown to impact HRQoL significantly, indicating the need for proactive evaluations and tailored interventions by healthcare providers. Future research should identify interventions that effectively improve HRQoL during the postpartum period.

## Introduction

Physical and psychological problems in women related to pregnancy and childbirth significantly influence their postpartum health-related quality of life (HRQoL; Al Rehaili et al., 2023). Moreover, HRQoL in postpartum women is significantly associated with their partners’ HRQoL (Ngai & Lam, 2021). Also, maternal mental health is crucial not only for the mother but also for the infant, as it is associated with childhood health and early emotional, relational, and social development (World Health Organization, 2022). Therefore, HRQoL provides a comprehensive assessment of the overall well-being of postpartum women and is a critical outcome measure in health care (Testa & Simonson, 1996).

Urinary incontinence (UI) is a common problem experienced by postpartum women. Approximately one-third of women suffer from UI within the first year after childbirth (Chang et al., 2021; Moossdorff-Steinhauser et al., 2021), which is a risk factor that negatively influences postpartum HRQoL (Martínez-Galiano et al., 2019; Triviño-Juárez et al., 2017). However, the respective associations between UI type and severity and HRQoL remain inconclusive. Although some studies have reported a significant relationship (Hatem et al., 2005), others have found no association (Handa et al., 2007).

In addition to UI, the overall prevalence of dyspareunia among postpartum women has been reported as 35% in the 12 months following childbirth (Banaei et al., 2021). Common forms of postpartum sexual dysfunction include loss of sexual desire, difficulties with arousal, lubrication problems, pain during intercourse, and challenges in achieving orgasm (O’Malley et al., 2023). These sexual dysfunctions can adversely affect HRQoL in women during the postpartum period (Rezaei et al., 2018). However, the effects of sexual dysfunction and specific sexual domains on HRQoL over time remain unclear.

In addition to physical and sexual health challenges, postpartum depression affects 10%–15% of women (Almuqbil et al., 2022), with depressive symptoms during the postpartum period found to impact both physical and mental HRQoL (de Tychey et al., 2008; Dennis, 2004). However, previous studies have focused primarily on the effects of depressive symptoms only, highlighting a need to examine how common issues such as UI, sexual dysfunction, and depressive symptoms interact and collectively influence postpartum HRQoL over time.

Although an increase in HRQoL in women between 6 and 12 weeks postpartum was reported in one study (Bahrami et al., 2014), comprehensive investigations of the changes in the physical and mental domains of HRQoL using a within-participants design with rigorous testing over the first year postpartum have only rarely been performed.

Postpartum women often face significant physical and psychological challenges that adversely affect their HRQoL. Common issues such as UI, sexual dysfunction, and postpartum depression are prevalent and negatively impact HRQoL (de Tychey et al., 2008; Dennis, 2004; Martínez-Galiano et al., 2019; Rezaei et al., 2018). How UI, sexual dysfunction, and depressive symptoms, in terms of both severity and type, impact HRQoL across the postpartum period remains unclear. Moreover, existing studies have not adequately explored how these factors interact and collectively influence HRQoL over time.

In response to these gaps and to improve the overall well-being of postpartum women, this study was developed to equip healthcare providers with the knowledge needed to effectively address the multifaceted challenges faced during the extended postpartum period. This prospective, four-time-point follow-up study was designed to investigate the association between HRQoL and, respectively, UI type; UI severity level; sexual dysfunction; the sexual function domains of desire, arousal, lubrication, orgasm, satisfaction, and pain; and depressive symptoms over the first 12-month postpartum period after adjusting for covariates. In addition, changes in HRQoL patterns between 1 and 12 months postpartum were explored to provide valuable insights into the dynamic nature of postpartum well-being.

## Methods

### Study Design

A prospective longitudinal design was employed, and data were collected at four time points: 4–6 weeks and 3, 6, and 12-months postpartum.

### Participants

The participants were recruited using convenience sampling from a hospital maternity unit. Inclusion criteria were postpartum women who were at least 18 years old, were literate in Traditional Chinese, and gave birth in the hospital’s maternity unit. Women with severe postpartum complications or complaints of tiredness were excluded. The minimum sample size calculation was performed using anticipated effect sizes for variables including UI, sexual dysfunction, depressive symptoms, and personal characteristics such as demographics, obstetric history, and childbirth information. The calculation assumed a significance level of .05 and a statistical power of 80%. Furthermore, a 15% dropout rate was incorporated into the estimation to account for potential attrition across the four time points.

### Procedure

A team of three trained data collectors—one full-time and two part-time—collected study data. To ensure data quality and consistency, all had received rigorous training, and data were checked regularly throughout the study. Data collection began in February 2016, and the final follow-up was completed in February 2020. During the face-to-face interviews held in the maternity unit, eligible participants were informed about the study and asked to read and sign a consent form before participating. Also, the participants were informed they could withdraw from this study at any time. Gift vouchers were provided to incentivize continued participation.

The participants completed the demographic data sheet in the maternity unit within three days of giving birth. Data on self-reported HRQoL, UI, sexual function, pain, and depressive symptoms were collected by mail at 4–6 weeks and 3, 6, and 12-months postpartum. Demographics, obstetric history, and childbirth information were obtained from participants' medical records and self-reports given during hospitalization after childbirth.

### Measurements

HRQoL was measured using the 36-item Short-Form Health Survey (SF-36), which is a reliable instrument for this purpose (Tseng et al., 2003; Ware & Sherbourne, 1992). Cronbach’s alpha coefficients ranged from .65 to .96 in prior studies (Brazier et al., 1992; Lu et al., 2003) and were between .78 and .80 at each time point in this study. Prior research demonstrated the SF-36’s construct validity among different age and health status groups (Brazier et al., 1992; Tseng et al., 2003). The SF-36 includes physical and mental component summaries (PCS and MCS). The four domains of the PCS include physical function, physical role, bodily pain, and general health, while the four domains of the MCS include validity, social function, role emotion, and mental health (Ware et al., 1993; Ware & Sherbourne, 1992). Questionnaire items are scored on a five-point scale, with each domain converted to a 0–100 scale on which higher scores indicate better health status or HRQoL (Ware & Sherbourne, 1992). The PCS and MCS scores were the sum of individual domain scores multiplied by each of the eight weights of PCS and MCS, respectively. These eight weights were adapted from the work of Ware (Ware et al., 1993; Ware & Sherbourne, 1992).

The International Consultation on Incontinence Questionnaire Urinary Incontinence Short Form (ICIQ-UI SF; Avery et al., 2004; Chang, Chen, Chang, et al., 2011) was used based on its good Cronbach’s alpha coefficient (>.8) in Chang, Chen, Chang, et al. (2011) and this study (.77–.85). The ICIQ-UI SF, which measures UI prevalence, severity, and interference with three scored questions (Avery et al., 2004), was used to evaluate the symptoms and effects of UI in this study. UI type (stress, urge, and mixed UI) was assessed using the self-diagnostic item: “When does urine leak?” (Avery et al., 2004; Rotar et al., 2009).

The Female Sexual Function Index (FSFI) was used to assess female sexual function in six domains: desire, arousal, lubrication, orgasm, satisfaction, and pain (Chang et al., 2009; Rosen et al., 2000). FSFI is a reliable instrument for evaluating sexual function in postpartum women (Chang, Chen, Lin, et al., 2011), and Cronbach’s alpha coefficient estimates of the FSFI were high (>.96) in a previous study (Chang et al., 2009) and were >.97 in this study. This scale comprises 19 items scored using a five-point Likert scale (Rosen et al., 2000). Each FSFI domain score was calculated by summing the scores of its corresponding items and multiplying by the domain-specific weighting factor. The total score was then obtained by summing all weighted domain scores. Self-reported total scores below 26.55 are interpreted as indicative of sexual dysfunction (Wiegel et al., 2005).

The Center for Epidemiologic Studies Depression Scale (CES-D) has high sensitivity and specificity for assessing depressive symptoms (Roberts & Vernon, 1983). This scale consists of 20 items rated on a four-point Likert scale (0=*never* and 3=*always*) ranging from 0 to 60 to assess the frequency of depressive symptoms (Chien & Cheng, 1985; Radloff, 1977). The alpha coefficient was high (>.85) in a previous study (Radloff, 1977) as well as in this study (.93–.94). Scale scores of 16 or higher in women are interpreted as indicative of depression (McDowell, 2006; Roberts & Vernon, 1983).

### Statistical Analyses

Descriptive analyses and statistical procedures were used to characterize the participants and the distribution of critical variables, including SF-36 (PCS and MCS), UI types, UI severity, UI interference, and FSFI function. Personal characteristics comprised time-dependent and fixed variables. Time-dependent variables included age, current body mass index, employment status, medical condition, feeding type, pain, UI type, UI severity, UI interference, CES-D, FSFI, and PCS and MCS of the SF-36. The fixed variables included marital status, educational level, personal income, household income, gestational age, spontaneous abortion, infertility experience, assisted reproduction, gravidity, parity, expected pregnancy, delivery method, degree of perineal laceration, newborn body weight, number of newborns, newborn care unit, and Apgar score.

Reliability of the UI, CES-D, FSFI, and SF-36 was assessed using Cronbach’s alpha coefficient. A paired *t* test was used to analyze longitudinal changes in the SF-36 scores across postpartum time points. Statistical significance was set at *p* <.05.

The outcome variables were the PCS and MCS of the SF-36 at four postpartum time points (1, 3, 6, and 12 months), which were used to analyze the factors associated with HRQoL. Personal characteristics were considered covariates. Univariate and multivariate generalized estimating equations and linear regression models were used to examine associations between the independent variables and HRQoL. Correlation coefficient analyses were employed to assess multicollinearity among independent variables. Those variables demonstrating significance (*p* <.05) in the univariate analysis were incorporated into the multivariate model. Significant variables in the multivariate analysis (*p* <.05) were identified as the associated factors. All of the statistical analyses were conducted using IBM SPSS Statistics Version 25.0 (IBM Corp., Armonk, NY, USA).

### Ethical Considerations

The research ethics committee of National Taiwan University Hospital (number 201412124RINA) reviewed and approved this research project. The study purpose and methods were clearly explained to the participants, all of whose information and data were anonymized and kept confidential.

## Results

Of the 1,045 participants, 613 completed the four time-point surveys and were included in the analyses. The mean age of all 613 participants was 34.1 (*SD* = 3.9) years, and the mean gestational age was 38.2 weeks (*SE* =2.0). Most were married (98.7%) and had attained a university education (61.3%). Approximately 60.0% were primiparas and 58.4% had vaginal births. The characteristics of the participants at each time point are presented in Table 1.

**Table 1:** Participant Characteristics at Four-Time Points and Their Associations With PCS and MCS During the First Year After Birth (*N*=613)

Item	M1	M3	M6	M12	PCS		MCS	
	*M*±*SE/n* (%)	*M*±*SE /n* (%)	*M*±*SE/n* (%)	*M*±*SE/n* (%)	Beta/95% CI	*p*	Beta/ 95% CI	*p*
Age (years)	34.1±3.9	34.3±3.9	34.6±3.9	35.0±3.9	−0.08 [−0.18, 0.03]	.158	0.13 [−0.04, 0.31]	.132
Body mass index (current BMI), kg/m^2^	23.2±3.0	22.8±3.2	22.4±3.3	22.1±3.3	−0.39 [−0.53, −0.26]	<.001**	−0.01 [−0.20, 0.19]	.942
Employment status ^a^								
Non−full-time	272 (44.7)	303 (49.6)	293 (48.1)	250 (41.2)	Ref.	—	Ref.	—
Full-time	337 (55.3)	308 (50.4)	316 (51.9)	357 (58.8)	0.78 [0.06, 1.51]	.035*	−0.22 [−1.29, 0.86]	.692
Medical condition ^a^								
No	490 (80.5)	490 (80.5)	487 (80.2)	493 (81.5)	Ref.	—	Ref.	—
Yes	119 (19.5)	119 (19.5)	120 (19.8)	112 (18.5)	−2.65 [−3.60, −1.71]	<.001**	0.03 [−1.32, 1.38]	.964
Feeding type								
Completely or mostly breastfeeding	406 (66.2)	363 (59.2)	274 (44.7)	123 (20.1)	Ref.	—	Ref.	—
Other feeding types	207 (33.7)	250 (40.8)	339 (55.3)	490 (79.9)	0.57 [−0.03, 1.17]	.063	0.34 [−0.48, 1.16]	.417
Pain, 6-point scale (0–5)	1.1±1.0	0.9±1.0	0.9±1.0	0.9±1.0	−2.79 [−3.08, −2.49]	<.001**	−1.32 [−1.72, −0.92]	<.001**
Marital status								
Single	8 (1.3)				Ref.	—	Ref.	—
Married	605 (98.7)				2.66 [0.41, 4.91]	.021^*^	1.90 [−1.97, 5.77]	.336
Educational level								
High school and below	30 (4.9)				Ref.	—	Ref.	—
University	373 (61.3)				1.67 [−0.46, 3.80]	.125	2.38 [−0.83, 5.58]	.146
Graduate and above	206 (33.8)				1.25 [−0.91, 3.42]	.257	2.53 [−0.76, 5.83]	.132
Personal income (USD/year)								
<3,333	95 (15.9)				Ref.	—	Ref.	—
3,333–16,666	183 (30.6)				0.90 [−0.51, 2.31]	.210	−1.10 [−3.39, 1.19]	.346
16,667–33,333	281 (46.9)				1.82 [0.51, 3.12]	.007*	−0.68 [−2.82, 1.45]	.531
>33,333	40 (6.7)				1.92 [−0.21, 4.06]	.077	1.56 [−1.41, 4.53]	.303
Household income (USD/year)								
<16,666	60 (10.0)				Ref.	—	Ref.	—
16,667– 33,333	167 (27.9)				0.96 [−0.64, 2.56]	.241	0.07 [−2.76, 2.89]	.963
33,334– 66,666	297 (49.6)				1.55 [0.06, 3.04]	.041*	0.63 [−2.09, 3.36]	.649
>66,666	75 (12.5)				2.20 [0.38, 4.02]	.018*	3.53 [0.37, 6.70]	.029*
Gestational age (week)	38.2±2.0				0.44 [0.23, 0.65]	<.001**	0.49 [0.17, 0.82]	.003*
Spontaneous abortion								
0	501 (82.4)				Ref.	—	Ref.	—
≥1	107 (17.6)				−1.41 [−2.50, −0.31]	.012*	0.77 [−1.14, 2.68]	.428
Infertility experience								
No	435 (72.6)				Ref.	—	Ref.	—
Yes	164 (27.4)				−1.00 [−1.97, −0.04]	.042*	−1.41 [−3.01, 0.18]	.081
Assisted reproduction								
No	457 (77.2)				Ref.	—	Ref.	—
Yes	135 (22.8)				−0.95 [−1.98, 0.08]	.070	−2.36 [−4.10, −0.62]	.008*
Gravidity								
Primigravida	250 (40.8)				Ref.	—	Ref.	—
Multigravida	363 (59.2)				−0.16 [−0.99, 0.66]	.695	2.04 [0.58, 3.50]	.006*
Parity					—	—		
Primiparous	364 (60.0)				Ref.	—	Ref.	—
Multiparous	243 (40.0)				0.20 [−0.65, 1.05]	.643	1.67 [0.22, 3.12]	.024*
Pregnancy expected								
No	52 (8.6)				Ref.	—	Ref.	—
Yes	550 (91.4)				−0.18 [−1.81, 1.46]	.833	−0.87 [−3.54, 1.81]	.526
Delivery method								
Vaginal delivery	340 (58.4)				Ref.	—	Ref.	—
Cesarean delivery	242 (41.6)				−0.88 [−1.73, −0.03]	.043*	−1.41 [−2.92, 0.09]	.066
Degree of perineal laceration								
No laceration	249 (42.5)				Ref.	—	Ref.	—
Mild	314 (53.6)				1.03 [0.16, 1.89]	.020*	1.60 [0.09, 3.11]	.038*
Severe	23 (3.9)				−0.36 [−2.35, 1.63]	.724	−2.18 [−5.44, 1.08]	.190
NBBW group	2985.8±502.1							
<2500 g	79 (13.4)				Ref.	—	Ref.	—
≥2500 g	511 (86.6)				0.67 [−0.65, 2.00]	.319	3.19 [0.87, 5.52]	.007*
Newborn number								
1	550 (93.1)				Ref.	—	Ref.	—
2	41 (6.9)				−1.90 [−3.70, −0.10]	.039*	−4.04 [−7.18, −0.91]	.012*
Newborn care unit								
Baby room or room in	454 (77.0)				Ref.	—	Ref.	—
Neonatal observation room	117 (19.8)				−0.67 [−1.71, 0.37]	.208	−2.49 [−4.42, −0.56]	.011*
Neonatal intensive care unit	19 (3.2)				−2.67 [−5.52, 0.18]	.066	0.46 [−3.89, 4.82]	.834
Apgar at 1 minute	8.7±0.8				0.49 [−0.09, 1.08]	.097	1.39 [0.56, 2.22]	.001*
Apgar at 5 minutes	9.0±0.3				1.03 [−0.32, 2.38]	.135	1.52 [−0.39, 3.43]	.119

*Note.* The time-dependent variables at 1, 3, 6, and 12 months following childbirth included age, body mass index, current medical condition, feeding type, and pain. PCS = physical component summaries; MCS = mental component summaries; M1 = 1-month postpartum; M3 = 3- months postpartum; M6 = 6- months postpartum; M12 = 12- months postpartum; *SE* = standard error; CI = confidence interval; Ref. = reference; NBBW = newborn birth weight.

^a^ Differences in participant numbers at each time point may result from non-response or data collection issues.

*p < .05. ^**^ *p* < .001.

Univariate analysis was used to examine the association between participant characteristics and PCS and MCS scores (Table 1). Current body mass index, employment, medical conditions, pain, marital status, personal and home income, gestational age, spontaneous abortion, infertility experience, delivery method, degree of perineal laceration, and number of newborns were found to be associated with PCS (Table 1). Pain, household income, gestational age, assisted reproduction, gravidity, parity, degree of perineal laceration, birth weight, number of newborns, newborn care unit, and Apgar score at 1 minute were found to be associated with MCS (Table 1). These significant variables served as the covariates for PCS and MCS scores in the multiple regression model.

**Figure 1:**
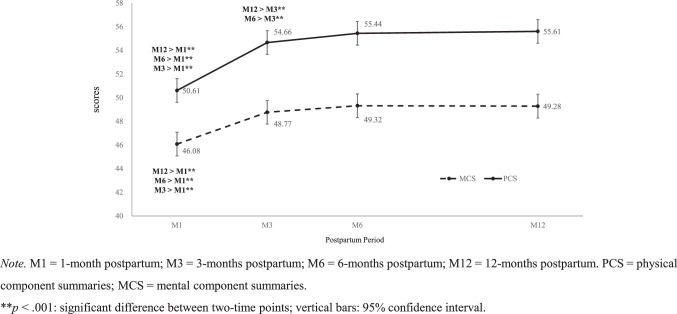
The Scores of PCS and MCS at Four-Time Points Over 12 Months Postpartum

The lowest PCS scores were found at 1 month postpartum (50.6±7.2; *p* <.001), and PCS scores increased significantly between 3 and 6 months and 3 and 12 months postpartum (all *p* ≤.001). However, no significant difference was noted in the PCS scores between 6 and 12 months postpartum (Table 2 and Figure 1).

**Table 2:** Distributions and Mean Differences for PCS and MCS Between Each Postpartum Time Point

Item	Mean±Standard Error				*p*					
	M1	M3	M6	M12	M1– M3	M1–M6	M1–M12	M3–M6	M3–M12	M6–M12
PCS	50.6±7.2	54.7±6.8	55.4±6.4	55.6±6.7	<.001**	<.001**	<.001**	<.001*	.001*	.528
MCS	46.1±10.8	48.8±10.9	49.3±10.7	49.3±10.2	<.001**	<.001**	<.001**	.080	.129	.916

*Note.* PCS = physical component summaries; MCS = mental component summaries; M1=1-month postpartum; M3=3 months postpartum; M6=6 months postpartum; M12=12 months postpartum.

^*^ *p* < .05. **p < .001.

Similarly, the lowest MCS scores were found at 1 month postpartum (46.1±10.8; *p*<.001). However, no significant differences were observed between MCS scores at 3 and 6 months, 3 and 12 months, and 6 and 12 months postpartum (Table 2 and Figure 1).

The scores and distributions of UI, sexual function, and depressive symptoms measured using the ICIQ-UI SF, FSFI, and CES-D at all four postpartum time points are given in Table 3. Approximately one-third (30.3%–34.3%) of the participants experienced UI during the first year after birth. Stress UI was the most frequent type (15.7%–21.9%), followed by urge and mixed types, with9.2%–10.7% of participants experiencing moderate to high severity. During the first three months postpartum, 85.7%–96.3% of the participants experienced FSFI dysfunction, and 72.5% continued to experience FSFI dysfunction throughout the first year postpartum.

**Table 3:** Distribution of Urinary Incontinence, Sexual Function, and Depressive Symptoms at Each Postpartum Time Point

Item	M1	M3	M6	M12
	*n* (%)/*M*±*SE*	*n* (%)/*M*±*SE*	*n* (%)/*M*±*SE*	*n* (%)/*M*±*SE*
UI type				
Any UI	185 (30.3)	189 (30.9)	187 (30.7)	208 (34.3)
Stress UI	96 (15.7)	112 (18.3)	119 (19.5)	133 (21.9)
Urge UI	47 (7.7)	44 (7.2)	33 (5.4)	40 (6.6)
Mixed UI	27 (4.4)	16 (2.6)	10 (1.6)	22 (3.6)
Other UI	15 (2.5)	17 (2.8)	25 (4.1)	13 (2.1)
UI severity				
Mild	123 (20.1)	140 (22.9)	130 (21.4)	141 (23.2)
Moderate & severe & very severe	64 (10.5)	64 (10.5)	56 (9.2)	65 (10.7)
UI interference	0.7±1.6	0.7±1.6	0.6±1.5	0.7±1.5
FSFI				
Sexual dysfunction (total scores ≤26.55)	577 (96.3)	520 (85.7)	480 (79.0)	442 (72.5)
Total scores	7.7±7.0	14.0±10.2	16.7±10.4	18.7±10.2
FSFI domains				
Desire	1.7±0.8	2.2±1.0	2.4±1.0	2.6±1.0
Arousal	0.7±1.4	1.9±1.9	2.4±2.0	2.7±1.9
Lubrication	0.8±1.6	2.2±2.2	2.8±2.3	3.3±2.2
Orgasm	0.7±1.5	2.1±2.2	2.6±2.2	3.0±2.1
Satisfaction	3.0±1.2	3.6±1.5	3.7±1.5	3.8±1.6
Pain	0.7±1.6	2.1±2.3	2.8±2.4	3.3±2.3
CES-D				
Depressive symptoms (total scores ≥16)	228 (37.2)	168 (27.4)	167 (27.2)	178 (29.1)
Total scores	14.1±10.4	11.5±9.9	11.5±9.5	11.7±9.7

*Note.* M1 = 1-month postpartum; M3 = 3-months postpartum; M6 = 6-months postpartum; M12 = 12-months postpartum; UI = urinary incontinence; FSFI = Female Sexual Function Index; CES-D = Center for Epidemiologic Studies Depression Scale.

### Factors Associated With Physical Quality of Life

In the univariate analysis, factors encompassing all domains of the FSFI, sexual dysfunction (score ≤26.55), and the high CES-D group (score ≥16) were found to be significantly associated with PCS. Also, UI, including mild and moderate-to-very severe UI, any UI, and urge UI, was found to be significantly associated with PCS (Table 4).

**Table 4:** Factors Associated With PCS and MCS Using Univariate and Multivariate Regression Analysis With a Generalized Estimating Equation

Item	PCS				MCS			
	Univariate		Multivariate		Univariate		Multivariate	
	Beta/ 95% CI	*p*	Beta/ 95% CI	*p*	Beta/ 95% CI	*p*	Beta/ 95% CI	*p*
Any UI								
No	Ref.	—	Ref.	—	Ref.	—		
Yes	−1.29 [−1.99, −0.58]	<.001**	1.45 [−1.21, 4.10]	.286	−0.51 [−1.41, 0.39]	.264	—	
Stress UI								
No	Ref.	—			Ref.	—		
Yes	−0.53 [−1.28, 0.22]	.164	—		0.21 [−0.73, 1.15]	.663	—	
Urge UI								
No	Ref.	—	Ref.	—	Ref.	—		
Yes	−1.31 [−2.40, −0.21]	.019^*^	−0.35 [−1.53, 0.84]	.566	−0.36 [−1.73, 1.00]	.601	—	
Mixed UI								
No	Ref.	—			Ref.	—		
Yes	−0.96 [−2.48, 0.57]	.219	—		−1.67 [−3.94, 0.60]	.148	—	
Other UI								
No	Ref.	—			Ref.	—		
Yes	−1.28 [−3.07, 0.52]	.163	—		−0.92 [−2.66, 0.83]	.304	—	
UI severity								
No	Ref.	—	Ref.	—	Ref.	—	Ref.	—
Mild	−0.90 [−1.64, −0.17]	.016^*^	−1.92 [−4.63, 0.79]	.165	−0.32 [−1.24, 0.60]	.497	−0.62 [−1.49, 0.25]	.165
Moderate & severe & very severe	−2.44 [−3.50, −1.38]	<.001**	−2.99 [−5.83, −0.14]	.040^*^	−2.08 [−3.40, −0.75]	.002^*^	−1.30 [−2.51, −0.10]	.033^*^
UI interference	−0.44 [−0.69, −0.18]	<.001**	NA		−0.42 [−0.70, −0.14]	.003^*^	NA	
FSFI domains								
Desire	1.11 [0.81, 1.42]	<.001**	0.31 [−0.11, 0.74]	.146	1.19 [0.78, 1.60]	<.001**	0.48 [−0.02, 0.99]	.060
Arousal	0.67 [0.54, 0.81]	<.001**	−0.03 [−0.52, 0.46]	.915	0.67 [0.48, 0.85]	<.001**	−0.25 [−0.80, 0.30]	.374
Lubrication	0.60 [0.49, 0.72]	<.001**	0.44 [0.00, 0.88]	.050^*^	0.53 [0.38, 0.69]	<.001**	0.26 [−0.22, 0.74]	.282
Orgasm	0.61 [0.49, 0.74]	<.001**	0.04 [−0.49, 0.56]	.892	0.57 [0.41, 0.73]	<.001**	−0.14 [−0.68, 0.40]	.617
Satisfaction	0.59 [0.38, 0.81]	<.001**	−0.43 [−0.74, −0.12]	.006^*^	1.21 [0.92, 1.50]	<.001**	0.61 [0.22, 1.00]	.002^*^
Pain	0.56 [0.45, 0.67]	<.001**	0.02 [−0.23, 0.26]	.891	0.49 [0.34, 0.64]	<.001**	0.07 [−0.24, 0.38]	.661
FSFI score								
High score group (>26.55)	Ref.	—	Ref.	—	Ref.	—	Ref.	—
Low score group (≤26.55, sexual dysfunction)	−2.60 [−3.24, −1.95]	<.001**	−0.84 [−1.73, 0.06]	.067	−2.70 [−3.68, −1.73]	<.001**	−0.44 [−1.58, 0.69]	.444
CES-D score								
Low score group (<16)	Ref.	—	Ref.	—	Ref.	—	Ref.	—
High score group (≥16, depressive symptoms)	−1.86 [−2.57, −1.16]	<.001**	−0.39 [−1.08, 0.30]	.267	−11.69 [−12.69, −10.68]	<.001**	−11.07 [−12.13, −10.00]	<.001**

*Note.* PCS = physical component summaries; MCS = mental component summaries; CI = Confidence interval; UI = urinary incontinence; Ref. = reference; NA = not applicable: UI interference was excluded from the independent variables for the multivariate generalized estimating equations, as there was multicollinearity between UI interference and UI severity; FSFI = Female Sexual Function Index; CES-D = Center for Epidemiologic Studies Depression Scale. The multiple generalized estimating equation model for PCS: The covariates, including body mass index, marriage, medical condition, gestation age, and pain, were entered into the generalized estimating equation model for PCS over 12 months after delivery. The independent variables were excluded from the multivariate generalized estimating equation because the *p-*value in univariate analysis was ≥.05. The multiple generalized estimating equation model for MCS: The covariates, including household income, multigravida, newborn care unit in neonatal intensive care unit, Apgar score at 1 minute, and pain, were entered into the generalized estimating equation model for MCS over 12 months after delivery. The independent variables were excluded from the multivariate generalized estimating equation because the *p*-value in univariate analysis was ≥.05.

^*^ *p* <.05. ^**^ *p* < .001.

After conducting a multivariate analysis controlling for the covariates, the sexual satisfaction and lubrication domains of the FSFI were found to be associated with PCS (β =−0.43, *p* =.006, β =0.44, *p* =.0497, respectively). Furthermore, moderate to very severe UI was shown to be negatively associated with PCS (β =−2.99, *p* =.04) but not with UI type (Table 4).

### Factors Associated With Mental Quality of Life

In the univariate analysis, factors including all domains of the FSFI, sexual dysfunction (FSFI score ≤26.55), and the high CES-D group (score ≥16) were found to be significantly associated with MCS. With regard to UI, only moderate to very severe UI was identified as significantly related to MCS (Table 4).

In the multivariate analysis, after controlling for the covariates, the sexual satisfaction domain of the FSFI was found to be associated with MCS (β =0.61, *p* =.002). Moreover, a significantly negative association was observed between the high CES-D group and MCS scores (β =−11.07, *p* <.001), and moderate to very severe UI was negatively associated with MCS (β =−1.3, *p* =.033; Table 4).

## Discussion

To the best of the authors’ knowledge, the association among sexual dysfunction, UI, and HRQoL has rarely been comprehensively and concurrently investigated within the same cohort during the first year postpartum. The results of this study highlight that the lowest physical and mental HRQoL scores during the first postpartum year were attained at one month postpartum. Also, while physical HRQoL was higher at both 6 and 12 months than at 3 months, mental HRQoL remained largely unchanged. Thus, moderate to very severe UI may predict worse physical and mental HRQoL, while UI type cannot. The sexual satisfaction domain of the FSFI was associated with physical and mental HRQoL, whereas the lubrication domain related positively to physical HRQoL only.

In this longitudinal study, moderate to very severe UI, rather than the UI type, emerged as a critical factor that negatively impacted both physical and mental HRQoL. This finding highlights the substantial burden moderate to severe UI places on postpartum women, disrupting their daily lives and overall well-being. For example, Chang et al. (2014) demonstrated that postpartum women with moderate to severe UI experienced heightened interference with daily activities, offering a credible explanation for the negative effects observed in this study on both physical and mental HRQoL. Findings that differ from this study have been reported in several cross-sectional studies. For example, Handa et al. (2007) concluded that neither the type nor the severity of UI was significantly associated with HRQoL, possibly due to differences from this study in terms of study design or sample characteristics. Conversely, Hatem et al. (2005) reported significant associations between both UI type and severity and HRQoL. Despite differences in research design and methodology, the mean age of participants in this study (34.1±3.9 years) was significantly higher than in Hatem and Handa’s studies (27.2–27.5 years), which may have also influenced patient UI experiences and impact on HRQoL via factors such as decline in pelvic floor muscle strength and elasticity, especially after pregnancy and childbirth (Xue et al., 2020). Consequently, further research is warranted to explore the effects of age on UI type and severity and their impact on HRQoL among postpartum women.

The results of the univariate analysis in this study, which revealed all domains of the FSFI to relate positively with physical and mental HRQoL during the first year postpartum, partially aligned with a previous cross-sectional study that identified four FSFI domains that influenced HRQoL positively (Rezaei et al., 2018). Moreover, the multivariate analysis findings were similar to those of Chang et al. (2019), which demonstrated an association between the satisfaction domain of the FSFI and mental HRQoL in middle-aged women (Chang et al., 2019). The findings of this study contribute significantly to the existing literature by highlighting the association between the satisfaction domain of the FSFI and both mental and physical HRQoL in postpartum women during the 12-month period following childbirth. This relationship may be understood through several underlying mechanisms. Physiologically, the postpartum recovery process often includes a gradual restoration of sexual function, as suggested by the findings in this study and corroborated by Chang et al. (2015). This recovery likely enhances physical HRQoL by improving bodily comfort, functionality, and overall health status.

In addition, the results of this study highlight the pivotal role of sexual satisfaction in shaping mental HRQoL. Beyond its physical implications, sexual satisfaction serves as a cornerstone for emotional intimacy within a partnership. This intimacy is not merely about physical closeness but also reflects mutual trust, understanding, and support, which are essential to psychological well-being. For postpartum women, who may experience heightened emotional vulnerability, the emotional reinforcement derived from a fulfilling sexual relationship can mitigate stress and foster resilience. Previous research supports this perspective, with Jeong et al. (2021) and Lokubal et al. (2021) underscoring how emotional and relational factors contribute significantly to mental HRQoL (Jeong et al., 2021; Lokubal et al., 2021). By integrating these insights, this study provides a holistic understanding of how sexual satisfaction influences postpartum HRQoL, offering valuable implications for healthcare interventions aimed at supporting women during this critical life stage.

However, the negative association found in this study between satisfaction and physical HRQoL in the multivariate regression analysis contrasts with the positive association observed in the univariate regression analysis. This discrepancy may be explained by the influence of the multiple independent variables considered simultaneously in the multivariate analysis, which may have altered the direction of the effect of sexual satisfaction on physical HRQoL.

Furthermore, the results indicate that higher lubrication domain scores on the FSFI predict better physical HRQoL in postpartum women. Postpartum women may experience symptoms such as vaginal dryness, thinning of the vaginal lining, and decreased vaginal lubrication during sexual activity. These symptoms are common during the postpartum period and breastfeeding due to sex hormone, especially estrogen, suppression (Millheiser, 2012). Therefore, our findings underscore the importance of lubrication as a sexual issue in postpartum women.

The analysis revealed that the high CES-D group (scores ≥16) scored relatively lower on the Mental Component Summary (MCS) but not on the Physical Component Summary (PCS) over the 12-month postpartum period. This finding underscores the substantial impact of depressive symptoms on mental HRQoL during the postpartum phase. The co-occurrence of depressive symptoms, anxiety, and fatigue, as highlighted in prior research (Kuo et al., 2012; Kuo et al., 2014), suggests these interconnected psychological stressors create a compounded burden on mental well-being. The absence of a significant effect on PCS may reflect the distinct ways in which psychological and physical domains are influenced during this period, emphasizing mental HRQoL as more directly affected by emotional and psychological states. The findings of this study were partially consistent with those of previous research in which postpartum women with depressive symptoms earned lower scores than their peers without depressive symptoms on, respectively, all domains of the SF-36 during the first eight weeks postpartum (de Tychey et al., 2008; Dennis, 2004) and both the PCS and MCS of the SF-12 during the first 24 weeks postpartum (Almuqbil et al., 2022). However, these differences may be attributable to variations in study design and/or analysis methods used. In addition to the different time periods investigated, our study identified the association between depression and HRQoL using multivariate regression analysis to adjust for covariates, while previous studies have primarily used *t* tests to examine differences in HRQoL (Almuqbil et al., 2022; de Tychey et al., 2008; Dennis, 2004).

In this study, the lowest PCS and MCS were observed at 1 month postpartum, highlighting the physical and emotional challenges that women faced during the early postpartum period. Physically, this initial period is marked by recovery from childbirth, which often includes vaginal bleeding, perineal pain, and fatigue from disrupted sleep patterns, as well as the demands of nighttime breastfeeding (Chang, Chen, Lin, et al., 2011; Chang et al., 2015; Henderson et al., 2019). These factors collectively contribute to diminished physical quality of life during the first month after delivery. From a psychological perspective, early postpartum is a time of significant hormonal fluctuations (Millheiser, 2012) that can exacerbate emotional instability. Moreover, sleep deprivation (Lin-Lewry et al., 2023; Manconi et al., 2024) and postpartum depression (Chang et al., 2018; Kuo et al., 2014) are prevalent challenges known to affect mental well-being negatively. Also, many women grapple with body image concerns (Chen & Chang, 2023) and the need to adjust to their new maternal roles (Ngai & Chan, 2012), further contributing to decreased mental quality of life. These findings emphasize the critical importance of providing targeted support to women during the first month postpartum that addresses both their physical recovery and psychological adjustment needs to mitigate the pronounced impact of childbirth on quality of life. In addition, the findings of this study echoed those of Bahrami et al. (2014), which reported scores across all domains of the SF-36 being higher at 12–14 weeks than at 6–8 weeks postpartum. However, Slavin et al. found no significant differences at 6 and 26 weeks postpartum in either physical or mental HRQoL scores (Slavin et al., 2019). In addition to the differences in tools employed to measure HRQoL, the different patterns of HRQoL change found in our and previous studies may be attributable to differences in observation period durations.

### Strengths and Limitations

This study has notable strengths. The large sample (613 participants) that completed surveys at four-time points during the first postpartum year facilitated the comprehensive examination of HRQoL in postpartum women and associated factors. Furthermore, using both univariate and multivariate generalized estimating equation methods enhanced the robustness of the findings.

However, some limitations must be acknowledged. First, recruiting postpartum women from one medical center only potentially constrains the generalizability of the results to the broader population. Second, data on social support and assistance during the postpartum period, which are critical factors influencing the HRQoL of postpartum women (Ngai & Lam, 2021), were not collected in this study. Thus, other potential moderating effects of social support may not have been considered in the reported outcomes. In future research, measures of social support should be incorporated to better understand the effect of this factor on the physical, sexual, and mental health factors influencing postpartum HRQoL. Third, the reliance on self-reported questionnaires to assess urinary incontinence, sexual function, and depression may not be sufficient for objective or diagnostic evaluation. Nonetheless, the rigorous verification of the reliability and validity of the instruments employed in this study helps ensure the accuracy of the collected data.

### Implications for Practice and Research

The first month after delivery was identified as the postpartum period with the lowest physical and mental HRQoL scores, highlighting the need for professional attention and thorough assessment of the physical and mental health of new mothers during this critical early postpartum phase. Moreover, the PCS score significantly increased between 3 and 6 months postpartum and remained relatively stable between 6 and 12 months, while the MCS score increased between 1 and 3 months postpartum and remained relatively stable afterward (between 3 and 12 months). These findings suggest early postpartum interventions implemented within the first two months and targeting both physical and mental health may be particularly effective. Future research should focus on identifying specific interventions that promote and sustain improvements in HRQoL throughout the postpartum period.

In this study, moderate to very severe UI, sexual satisfaction, and lubrication issues were identified as important concerns affecting HRQoL in postpartum women. Therefore, proactive evaluations and tailored interventions by healthcare providers are recommended. These interventions may include advice on lubricant use to manage vaginal dryness and encouragement of pelvic floor muscle exercises to address postpartum UI problems (Nguyen et al., 2024). Furthermore, aromatherapy may positively impact depression in postpartum women (Tsai et al., 2020). Future studies should investigate the combined effects of aromatherapy and targeted interventions on the significant issues of postpartum physical and mental HRQoL to promote the provision of holistic, effective postpartum care.

### Conclusions

The postpartum women in this study reported the lowest physical and mental HRQoL scores during the first postpartum month; physical and mental HRQoL were identified as negatively influenced by moderate to very severe UI; sexual satisfaction was identified as positively associated with physical and mental HRQoL; and lubrication was identified as positively associated with physical HRQoL only.

Healthcare providers should assess key factors associated with HRQoL, including UI severity, sexual satisfaction, lubrication, and depressive symptoms, and provide ongoing, targeted consultations and preventive strategies.
